# Social relationships and HRQL: A cross-sectional survey among older Italian adults

**DOI:** 10.1186/1471-2458-8-348

**Published:** 2008-10-03

**Authors:** Antonio Giulio de Belvis, Maria Avolio, Lorella Sicuro, Aldo Rosano, Elide Latini, Gianfranco Damiani, Walter Ricciardi

**Affiliations:** 1 Department of Public Health and Preventive Medicine, Catholic University 'Sacro Cuore', Rome, Italy; 2 Italian Institute of Social Medicine, Rome, Italy; 3 Agency of Public Health, Lazio Region, Rome, Italy

## Abstract

**Background:**

The aim of this study is to investigate the association between social relationships and Health Related Quality of Life (HRQL) among the elderly in Italy.

**Methods:**

A sample of 33,744 Italian residents, representing the non-institutionalised population aged 60 years and over was extracted from the national ISTAT cross-sectional survey during 1999–2000. HRQL was measured with the SF-12, from which the Physical Component Score (PCS) and Mental Component Score (MCS) were obtained. Data were subjected to descriptive analysis and multiple logistic regression models with adjustment for the main confounders.

**Results:**

Our analysis shows a gradient in PCS and MCS among the terziles in seeing/meeting "friends" and "family" and, for PCS, a North-South gradient among the Italian regions. Females, the elderly who reported a lower household income, those who spent less time in recreational and religious activities, who lived too far from their relatives and had few relationships with friends and relatives, were significantly less likely to have an MCS above the median value. For PCS, an increase in HRQL was likely to be associated with a higher educational level, while lower PCS scores were associated with: age 75+, inadequate household income, unmarried status, infrequency of seeing/meeting friends, too high a mean distance from own home to relatives' homes, lack of leisure time spent in recreational activities, living in the Centre-South of Italy, chronic diseases, reduced autonomy, and use of drugs during the previous two days. Significant interactions between suffering from one chronic disease and the use of drugs were also found for both MCS and PCS.

**Conclusion:**

Some dimensions of social relationships were significantly associated with HRQL. These findings are crucial for devising welfare strategies at both the regional and the European level, i.e. in countries such as Italy where the primacy of family support of the elderly has declined in recent years.

## Background

Social relationships are defined as social structures made up of contact bonds among individuals or groups of relatives, colleagues, friends and neighbours. These relationships are based upon reciprocal trust [1,2]. Social networks may differ in composition, number of members, frequency of contact and also geographic proximity [3], and could be a crucial for an individual's way of life and represent a dimension of social capital that can influence reciprocal exchanges of support and knowledge.

A huge literature corroborates the relationship between social relationship and health status in the elderly [4-10] and poor social networks/relationships are likely to be associated with worse physical and mental health status [11,12].

In previous studies, different definitions, classifications, analyses of social relationships and their influence on health have been proposed. Among these, a recent review carried out by Berkman and Glass proposed a "cascade" model by which social and cultural characteristics determine the structure and shape of a social network [8], supporting – in turn – functional aspects that may influence health status via psychological and physiological pathways. This model was tested in a Mediterranean country by including psychosocial aspects related to social integration [13].

As in other Mediterranean countries, family/neighbour networks in Italy have long been essential for solidarity/support of the elderly. In recent years, the role of the family has changed, in Italy as elsewhere, because of fragmentation into more unstable and smaller structures. This changing role has generated a greater need for external public and private support [14] to handle the high percentage of elderly people in the Italian population (19.7% 65+ in 2001), and the percentage of elderly living alone (22.5%) or in long-term care [15,16]. To date, only a few studies have evaluated the relationship between social networks and perceived health status among the elderly in Mediterranean countries [17].

In recent years, the Italian health system has also undergone devolution from a national to a regional level. Since the late 1990s, this process has also involved health care delivery, with impacts on the quality of care, equity of access and health care status itself [18].

The aim of this study is to investigate the association between social relationships and Health Related Quality of Life (HRQL) among a sample of 33,744 individuals aged 60 years or over living in Italy in 1999–2000 [19].

## Methods

### Study design and respondents

Data are drawn from the survey "Health status of the population and use of health services – years 1999–2000", a national cross-sectional survey carried out every five years by the National Institute of Statistics (ISTAT) [19], that supplies information about perceived health status, disease symptoms, chronic disability conditions and social determinants of health on a national sample of 52,332 households, with 140,011 residents in 1,463 centres in Italy [20-22]. The survey is composed of two different questionnaires. The first investigates socio-demographic characteristics, cultural and economic conditions and region of residence. The second is self-compiled and gives information about HRQL, lifestyle, access to/use of health care services and non-institutional support systems. We focused on a sample of 33,744 elderly people residing in Italy (24% of the whole survey sample), representative of the non-institutionalised population aged 60 years and over in 1999–2000.

### Study variables

HRQL was measured using SF-12, from which a Physical Component Score (PCS) and a Mental Component Score (MCS) were obtained [23]. Data on social relationships were collected from questions about marital status (married vs. single, separated, divorced, widowed), living alone (yes/no), frequency of visiting/seeing relatives (sons/daughters, parents, brothers, nephews and other relatives) and friends, whether the average distance between own home and relatives' homes was considered too long by the Respondent (yes/no), and leisure time spent in religious and recreational activities (yes/no). In particular, "frequency of visiting family" and "frequency of visiting friends" scores were divided into terziles, the first representing the lowest frequency rate and the third the highest.

Information was also obtained on socio-demographic variables (age: 60–74, 75+; gender: male, female); geographical area (North West: Piemonte, Valle d'Aosta, Liguria, Lombardia; North East: Trentino Alto Adige, Veneto, Friuli Venezia Giulia, Emilia Romagna; Centre: Marche, Toscana, Umbria, Lazio; South: Campania, Abruzzo, Molise, Puglia, Basilicata, Calabria; Main Islands of Italy: Sicilia, Sardegna); educational level (no formal education, primary, secondary, university); self-assessment of household-income (good, adequate, inadequate, absolutely inadequate); lifestyle [tobacco use (smoker, former smoker, non-smoker); physical activity (yes, no)]; medical conditions [chronic diseases diagnosed by the General Practitioner (none, one chronic disease, more than one chronic disease, including: diabetes mellitus, hypertension, chronic bronchitis, acute myocardial infarction, angina pectoris, other heart diseases, cataracts, osteoarthritis, malignant neoplasms, Alzheimer's, Parkinson's, epilepsy and other memory disorders); occurrence of disease during the previous four weeks (yes, no); reduced autonomy because of chronic diseases (yes sometimes, yes often, no)]; access to health-care services [use of drugs in the previous two days (yes, no, unknown); medical consultation during the past four weeks (yes, no)].

### Data analysis

Results obtained on scales in the SF-12 questionnaire received a numerical score, which was standardized and ranked on a scale from 0–100, higher scores indicating a better self-reported health status [24].

PCS and MCS were categorized as dichotomous variables by considering values above and below the average, respectively. Initially, each relationship was assessed by univariate analysis. Multiple logistic regression was performed using two different models to verify the influence on MCS and PCS, respectively, of socio-demographic, lifestyle, social network, geographical area and health care service access/use as independent variables; all explicatory variables were taken as models. When an Odds Ratio (O.R.) is significantly higher than 1, the variable is likely to be associated with a higher score; when it is significantly less than 1, with a lower score.

We tested for collinearity by several diagnostic measures including Variance Inflation Factors and tolerance [25].

To test for interactions, we added the interactions one at a time to the main effects model and contrasted the likelihoods of the models including and excluding the interaction term using the Log-likelihood ratio test. If this proved statistically significant, we included the interaction terms in the final model [26]. Analysis was performed using SPSS Version 13.

## Results and discussion

Participation in the survey was enhanced by sending a letter with a clear introduction to the survey and by activating a toll-free number to provide information to interviewees [21]. The participation rate was 86.6% and the response rate 96.9%. The main reasons for not participating were: non-traceable household (51.8%) and refusal to participate (40.8%). Two main reasons for not responding were: refusal (0.5%), absence of the responder (0.2%) [19].

Descriptive analysis (Table 1) shows gradients in PCS and MCS among the terziles in seeing/meeting "friends" and "family". For PCS, our analysis showed a North-South gradient among the Italian regions.

**Table 1:** Descriptive statistics of the sample, by Physical (PCS) and Mental (MCS) Score Components

	**Variables**		**PCS**	**MCS**
**SOCIO-DEMOGRAPHIC VARIABLES**	***Age***	60–74	45.24 (10.50)	48.20 (10.82)
		75+	37.72 (11.51)	45.15 (12.07)
	***Gender***	male	44.68 (10.97)	48.92 (10.58)
		female	41.42 (11.49)	45.90 (11.71)
**CULTURAL-ECONOMIC VARIABLES**	***Educational level***	no formal education	38.15 (11.45)	44.60 (11.96)
		primary	43.65 (11.04)	47.70 (11.12)
		secondary	47.60 (9.94)	49.73 (10.21)
		university	48.57 (9.59)	50.46 (9.83)
	***Self assessment of household income***	good	47.49 (10.14)	50.87 (9.81)
		adequate	43.94 (11.08)	48.41 (10.74)
		inadequate	40.38 (11.57)	44.80 (11.96)
		absolutely inadequate	37.62 (11.61)	40.18 (12.85)
**LIFESTYLE**	***Tobacco use***	smoker	46.15 (10.20)	48.30 (10.95)
		former smoker	43.60 (11.20)	48.09 (11.02)
		non-smoker	41.88 (11.53)	46.65 (11.49)
	***Regular physical activities***	no	41.83 (11.61)	46.56 (11.57)
		yes	47.24 (9.14)	50.08 (9.70)
**SOCIAL NETWORKS**	***Marital status***	married	44.16 (10.93)	48.11 (10.90)
		unmarried	40.71 (11.78)	45.79 (11.85)
	***Living alone***	no	43.39 (11.24)	47.67 (11.11)
		yes	41.05 (11.66)	45.73 (11.89)
	***Distance too long between own home and relatives' home***	no	43.26 (11.29)	47.67 (11.12)
		yes	41.14 (11.62)	45.37 (11.97)
	***Frequency of seeing/meeting friends***	I terzile	38.93 (12.02)	43.42 (12.49)
		II terzile	42.18 (11.29)	46.89 (11.23)
		III terzile	44.69 (10.79)	48.80 (10.58)
	***Frequency of seeing/meeting family***	I terzile	41.72 (11.81)	45.62 (12.07)
		II terzile	42.22 (11.49)	47.02 (11.31)
		III terzile	43.89 (10.98)	48.27 (10.77)
	***Leisure time for spiritual activities in the last three months***	no	42.4 (11.54)	46.87 (11.49)
		yes	45.70 (9.90)	49.51 (9.96)
	***Leisure time for recreational activities in the last three months***	no	41.65 (11.51)	46.45 (11.60)
		yes	48.38 (8.91)	50.81 (9.15)
**GEOGRAPHICAL AREA**	***Regional aggregate***	North-West	44.81 (10.84)	48.10 (10.92)
		North-East	44.14 (11.13)	48.37 (11.18)
		Centre	43.15 (11.30)	46.95 (11.52)
		South	41.09 (11.38)	46.23 (11.26)
		Islands	41.02 (12.01)	46.64 (11.83)
**MEDICAL CONDITION AND HEALTH CARE SERVICES ACCESS/USE**	***Any disease in the last four weeks***	no	46.51 (10.16)	49.69 (10.00)
		yes	37.94 (11.08)	43.93 (12.14)
	***Reduced autonomy because of chronic disease***	no	45.7 (9.91)	48.97 (10.31)
		yes, sometimes	34.19 (9.47)	42.66 (11.99)
		yes, often	28.71 (8.82)	37.95 (12.48)
	***Chronic medical condition***	none	48.72 (9.16)	50.87 (9.23)
		one chronic diseases	44.18 (10.52)	48.37 (10.58)
		more than one chronic diseases	37.59 (11.09)	43.72 (12.21)
	***Drugs in the last two days***	no	48.99 (8.61)	51.38 (8.62)
		yes	40.05 (11.39)	45.33 (11.89)
		I don't know	42.93 (11.15)	47.29 (10.89)
	***Medical consultation***	no	45.49 (10.58)	48.93 (10.51)
		yes	38.97 (11.41)	44.73 (12.00)

(Mean and SD)

The regression model was used to test the relationship between a group of selected explanatory variables and both physical and mental health. Statistical significance was attained on the Physical and Mental health scores above and below the median values. For mental health (Table 2), females, the elderly who reported lower household income, those who spent less time in recreational and religious activities, those who lived too far from their relatives and had few relationships with friends and relatives and those living in the South rather than in the North West of Italy, were significantly less likely to have an MCS above the median value. The analysis also demonstrated a poorer MCS in respondents with rather than without one or more chronic diseases, while the occurrence of disease during the previous 4 weeks was likely to be associated with an MCS below the median value. In this model, the value of the Hosmer-Lemeshow goodness-of-fit statistic [χ^2 ^= 5.601, degrees of freedom (df) = 8, p = 0.692] confirmed that the model fitted the data quite well.

**Table 2:** Logistic regression model relating groups of variables and MCS

			**MCS**		
			**O.R**	**95.0% C.I.**	
				**Lower**	**Upper**
**SOCIO-DEMOGRAPHIC VARIABLES**	***Age***	60–74	1		
		75+	1.032	0.976	1.091
	***Gender***	male	1		
		female	0.705**	**0.660**	**0.754**
**CULTURAL-ECONOMICS VARIABLES**	***Self-assessment of household income***	good	1		
		adequate	0.843*	**0.723**	**0.984**
		inadequate	0.596**	**0.509**	**0.698**
		absolutely inadequate	0.405**	**0.327**	**0.503**
	***Educational level***	university	**1**		
		no formal education	0.780*	**0.668**	**0.911**
		primary	0.861*	**0.743**	**0.998**
		secondary	0.992	0.843	1.167
**LIFESTYLE**	***Regular physical activities***	yes	1		
		no	0.899*	**0.846**	**0.955**
	***Tobacco use***	non-smoker	1		
		smoker	0.861**	**0.797**	**0.930**
		former smoker	1.012	0.951	1.077
**SOCIAL NETWORK**	***Living alone***	no	1		
		yes	0.980	0.835	1.151
	***Marital status***	married	1		
		unmarried	0.905	0.794	1.031
	***Leisure time for spiritual activities in the last three months***	yes	1		
		no	0.815**	**0.761**	**0.873**
	***Leisure time for recreational activities in the last three months***	yes	1		
		no	0.820**	**0.767**	**0.876**
	***Distance too long between own home and relatives' home***	no	1		
		yes	0.863**	**0.812**	**0.917**
	***Frequency of seeing/meeting friends***	III terzile	1		
		I terzile	0.656**	**0.612**	**0.702**
		II terzile	0.871**	**0.826**	**0.918**
	***Frequency of seeing/meeting family***	III terzile	1		
		I terzile	0.828**	**0.779**	**0.879**
		II terzile	0.927*	**0.876**	**0.981**
**GEOGRAPHICAL AREA**	***Regional aggregate***	North-West	1		
		North-East	1.149**	**1.069**	**1.236**
		Centre	0.991	0.919	1.068
		South	0.912*	**0.850**	**0.979**
		Islands	1.037	0.948	1.134
**MEDICAL CONDITIONS AND HEALTH CARE SERVICES ACCESS/USE**	***Any disease in the last four weeks***	no	1		
		yes	0.658**	**0.626**	**0.692**
	***Reduced autonomy because of chronic disease***	no	1		
		yes, sometimes	0.622**	**0.572**	**0.677**
		yes, often	0.349**	**0.318**	**0.383**
	***Medical consultation***	no	1		
		yes	0.799**	**0.760**	**0.841**
	***Chronic medical condition***	none	1		
		one chronic disease	0.752**	**0.685**	**0.826**
		more than one chronic disease	0.509**	**0.446**	**0.579**
	***Drugs in the last two days***	no	1		
		yes	0.598**	**0.547**	**0.654**
		I don't know	0.553	0.277	1.104
**INTERACTIONS**	***Gender (male) by marital status (married)***		1		
	***Gender (female) by marital status (unmarried)***		1.116	0.957	1.302
	***Gender (male) by living alone (no)***		1		
	***Gender (female) by living alone (yes)***		1.061	0.883	1.274
	Chronic medical condition (none)*drugs in the last two days (no)		1		
	Chronic medical condition (one chronic disease)*drugs in the last two days (yes)		1.198*	**1.058**	**1.357**
	Chronic medical condition (one chronic disease)*drugs in the last two days (I don't know)		0.894	0.270	2.965
	Chronic medical condition (more than one chronic disease)*drugs in the last two days (yes)		1.262*	**1.085**	**1.467**
	Chronic medical condition (more than one chronic disease)*drugs in the last two days (I don't know)		1.252	0.401	3.908

* p < 0.05; **p < 0.001

For PCS (Table 3), an increase in SF-12 scores was likely to be associated with a higher educational level. Lower PCS scores were associated with: 75 years of age or over; inadequate household income; unmarried status; frequency of seeing/meeting friends in the second and first terzile rather than the third; too high a mean distance from own home to relatives' homes; lack of leisure time spent in recreational activities; living in the Centre, the South or in the main Islands of Italy; having one or more chronic diseases; occurrence of disease during the previous 4 weeks; reduced autonomy because of chronic disease; and use of drugs during the previous two days. The value of the Hosmer-Lemeshow goodness-of-fit statistic (χ^2 ^= 10.481, DF= 8, p: 0.233) again indicates that this model fits the data quite well.

**Table 3:** Logistic regression model relating groups of variables and PCS

			**PCS**		
			**O.R**	**95.0% C.I.**	
				**Lower**	**Upper**
**SOCIO-DEMOGRAPHIC VARIABLES**	***Age***	60–74	1		
		75+	0.510**	**0.479**	**0.543**
	***Gender***	male	1		
		female	0.868	0.746	1.009
**CULTURAL-ECONOMICS VARIABLES**	***Self-assessment of household income***	good	1		
		adequate	0.791*	**0.660**	**0.947**
		inadequate	0.579**	**0.481**	**0.697**
		absolutely inadequate	0.507**	**0.397**	**0.649**
	***Educational level***	university	1		
		no formal education	0.409**	**0.339**	**0.493**
		primary	0.550**	**0.459**	**0.659**
		secondary	0.809*	**0.664**	**0.986**
**LIFESTYLE**	***Regular physical activities***	yes	1		
		no	0.767**	**0.716**	**0.821**
	***Tobacco use***	non-smoker	1		
		smoker	1.131*	**1.036**	**1.236**
		former smoker	1.026	0.955	1.101
**SOCIAL NETWORK**	***Living alone***	no	**1**		
		yes	1.279**	**1.168**	**1.401**
	***Marital status***	married	1		
		unmarried	0.891*	**0.821**	**0.967**
	***Leisure time for spiritual activities in the last three months***	yes	1		
		no	0.917	0.810	1.038
	***Leisure time for recreational activities in the last three months***	yes	1		
		no	0.679**	**0.629**	**0.733**
	***Distance too long between own home and relatives' homes***	no	1		
		yes	0.893*	**0.834**	**0.957**
	***Frequency of seeing/meeting friends***	III terzile	**1**		
		I terzile	0.765**	**0.707**	**0.828**
		II terzile	0.857**	**0.807**	**0.911**
	***Frequency of seeing/meeting family***	III terzile	**1**		
		I terzile	0.965	0.900	1.034
		II terzile	0.983	0.921	1.050
**GEOGRAPHICAL AREA**	***Regional aggregate***	North-West	1		
		North-East	0.966	0.889	1.049
		Centre	0.909*	**0.834**	**0.990**
		South	0.648**	**0.598**	**0.703**
		Islands	0.683**	**0.617**	**0.757**
**MEDICAL CONDITIONS AND HEALTH CARE SERVICES ACCESS/USE**	***Any disease in the last four weeks***	no	1		
		yes	0.425**	**0.402**	**0.450**
	***Reduced autonomy because of chronic disease***	no	1		
		yes, sometimes	0.248**	**0.211**	**0.291**
		yes, often	0.149**	**0.123**	**0.179**
	***Medical consultation***	no	1		
		yes	0.670**	**0.596**	**0.753**
	***Chronic medical condition***	none	1		
		one chronic disease	0.523**	**0.466**	**0.587**
		more than one chronic disease	0.273**	**0.235**	**0.317**
	***Drugs in the last two days***	no	**1**		
		yes	0.375**	**0.337**	**0.418**
		I don't know	0.666	0.289	1.537
**INTERACTIONS**	***Leisure time for recreational activities in the last three months (yes) by gender (male)***		1		
	***Leisure time for recreational activities in the last three months (no) by gender (female***		0.882	0.755	1.031
	Reduced autonomy because of chronic disease (no)* gender (male)		1		
	***Reduced autonomy because of chronic disease (yes, sometimes) by gender (female)***		0.873	0.699	1.090
	***Reduced autonomy because of chronic disease (yes, often) by gender (female)***		0.624*	**0.470**	**0.829**
	Chronic medical condition (none) * medical consultation (no)		1		
	***Chronic medical condition (one chronic disease) by medical consultation (yes)***		0.953	0.821	1.106
	***Chronic medical condition (more than one chronic disease) by medical consultation (yes)***		1.062	0.920	1.226
	Chronic medical condition (none)*drugs in the last two days (no)		1		
	Chronic medical condition (one chronic disease)*drugs in the last two days (yes)		1.573**	**1.361**	**1.819**
	Chronic medical condition (one chronic disease)*drugs in the last two days (I don't know)		0.588	0.160	2.167
	Chronic medical condition (more than one chronic disease)*drugs in the last two days (yes)		1.663**	**1.402**	**1.972**
	Chronic medical condition (more than one chronic disease)*drugs in the last two days (I don't know)		0.660	0.163	2.677

* p < 0.05; **p < 0.001

Significant interactions between "one chronic disease or more than one chronic disease" and the "use of drugs during the previous two days" were found for both MCS and PCS, while there was a significant interaction between "being female" and "often having reduced autonomy because of chronic disease" only for PCS.

## Conclusion

We characterized social relationships among the elderly in Italy by investigating domains dealing with "structural" items (living alone, marital status, average distance from relatives' homes), with contacts (frequency of contacts with friends and relatives) and with participation in recreational and religious activities. Therefore, we assessed the association between social relationships and HRQL [27,28]. We found that some variables are significantly associated with both PCS and MCS.

Our study is the first in Italy, and one of the few in the Mediterranean area, to evaluate the influence of social networks on self-reported health status among the elderly, and has added new findings to previous research [17,29]. Our sample was mostly composed of married elderly people not living alone; 80.5% of the sample fulfilled these two conditions, confirming the findings of other studies previously conducted in Mediterranean countries [30].

The association between being 75 or older and a weakening self-estimated PCS has been demonstrated in other studies [29].

The role of self-assessment of household income and educational level is strongly evident. This would confirm other findings indicating that educational level may be used as a proxy for socio-economic conditions: belonging to lower social classes is likely to be associated with weaker ties of social and affective reciprocity, even if a decline in social network affects the quality of life in the elderly more dramatically [31,32].

We found that a lack of regular physical activity was likely to be associated with lower PCS and MCS scores. This seems quite self-explanatory [29].

As regards social relationships, we found significant associations between spending less leisure time in recreational activities, too long an average distance from relatives' homes, fewer contacts with friends and lower scores in PCS and MCS, regardless of other socio-economic and disease determinants [27].

Moreover, living in the Southern rather than the North Western regions of Italy is likely to be associated with lower PCS and MCS scores. These findings confirm that the devolution of health care delivery in Italy has to face different impacts on quality of care, equity of access and health care status itself, as other studies have recently demonstrated [17,33].

We also found a significant association between suffering from one or more chronic diseases, reduced autonomy because of chronic disease [34], occurrence of disease during the previous four weeks, medical consultations and drug use and a lowering of MCS and PCS scores. These findings are corroborated by the significant interactions between "one or more chronic diseases" and "use of drugs during the previous two days" for both MCS and PCS. Such associations and interactions also seem to be quite self-explanatory.

Smoking is also likely to be associated with a higher PCS. MCS notwithstanding, smoking habits among the elderly may be considered a proxy for relatively good self-rated physical health status and autonomy [35]. This could also explain the association between "living alone" and a PCS score above the median value [36].

On the other hand, being unmarried or being widowed/divorced is likely to be associated with a lower PCS, thus confirming other findings in Mediterranean countries [29,33].

Furthermore, as social integration should be distinguished from loneliness, a comparative study carried on 1992–1994 in two regions of Netherlands and Italy showed that on the average older adults in Tuscany are lonelier than adults in Netherlands [37].

In addition, there was a significant interaction between "being female" and "often having reduced autonomy because of chronic disease", and a decline in the physical dimension of perceived quality of life. This difference between men and women in the impact of social relationships and, among women, with those suffering from chronic diseases, appears to be coextensive with social isolation, a known risk factor in the quality of life [38].

We found a significant association between spending leisure time in religious activities, more frequent contacts with relatives, and a higher MCS score. A protective effect of attending spiritual activities on health status has previously been reported in the literature [39].

On the other hand, leisure time spent in cultural and religious activities needs additional psycho-physical functional capabilities [29]. The evidence that living alone has a protective effect on PCS in HRQL needs to be confirmed.

Our findings confirm that more frequent social relationships are likely to have protective effects on self-perceived mental and physical health status, corroborating the view that such relationships are likely to be associated with lower incidences of depression and anxiety.

Owing to the original database design, we focused on measures of total social contacts and did not disaggregate the interpersonal contacts inside kin- or non-kin networks [40]. This limitation, which would not reinforce the role of social choices among the elderly and the effects of confidants in social relationships, is likely to be less strong when we consider that relatively high levels of social contacts in Italian society are centred on family life, and that in our framework contacts are part of social relationships as well as other structural and participative dimensions.

Cross-sectional data describe the association between "exposure" (social relationships) and "outcome" (HRQL), but in analysing such a relationship it would be useful to determine causation. Social relationships and HRQL among the elderly are reciprocally connected, particularly over an extended time span, so that loneliness and lack of social support may become both cause and consequence of disease [41]. This is particularly relevant when an ecological study with this design is solicited to investigate new public health issues, to generate hypotheses about their causation and to focus on the necessary actions. In addition, when people become fragile and require more support, the network size and frequency of relationships would increase [42]. Therefore, longitudinal studies are necessary to clarify the relationships between HRQL and the role of social networks among the elderly, and the associations with illnesses, disabilities, and the access to/use of health care services [43,44].

We used SF-12 to assess HRQL. This instrument focuses on dimensions of quality of life based on health and health care. Therefore, it is not possible to assess whether associations with other dimensions of HRQL do not exist or are not detectable by SF-12 [21].

In addition, recall bias may affect self-assessment of quality of life, even if the self-declaration of chronic disease is confirmed by a GP's diagnosis. Nevertheless, such self-rated measures have been shown to be significant predictors of morbidity [45], as recently confirmed by another survey in Italy [46].

Another limitation of the ISTAT questionnaire is its inability to describe all the components of social relationships (i.e. public involvement or size and quality of social interactions). However, the variables do provide information on the existence of social ties and frequency of meeting/seeing friends and relatives, as they presuppose a minimum level of functional capacity.

In addition, our one-dimensional epidemiological measures, taken from items designed for other purposes or from available stocks of post hoc measures, are therefore not exhaustive, and recent researchers have applied multi-dimensional measures of social networks among the elderly [47]. Social networks influence old people's behaviour, self-care and demand for/access to health care [48]. They represent a key resource of social capital, one of the most widely-accepted social determinants of health status and health promotion. This concept entails implications for public health choices [49].

In Italy, as well as in Southern Europe, the role of family and the traditional family support for the elderly have been affected by demographic, economic and social changes. This is an important issue to consider when assessing welfare policies orientated towards the elderly, as well as Beveridge systems (including UK, Sweden, Denmark, Finland and Italy, and characterized by free-of-charge health services with mainly public providers). This suggests the need to enhance the role of non-institutional interventions of networks for the elderly, including those involving the elderly as support-givers [50], differently shaped according to the characteristics of each region.

## Competing interests

The authors declare that they have no competing interests.

## Authors' contributions

AGdB: conceived of the study, participated in its design and drafted the manuscript; MA: performed the statistical analysis and drafted the manuscript; LS: performed the statistical analysis; AR: designed the study and conducted the statistical analysis; GD: conceived of the study and participated in its design; EL: participated in the study design; WR: conceived of the study and participated in its coordination. All authors have read and approved the final manuscript.

## Pre-publication history

The pre-publication history for this paper can be accessed here:


